# Cryo-EM reveals remodeling of a tandem riboswitch at 2.9 Å resolution

**DOI:** 10.21203/rs.3.rs-6422592/v1

**Published:** 2025-05-02

**Authors:** Nathan Jespersen, Jigneshkumar Dahyabhai Prajapati, Ankush Singhal, Karissa Y. Sanbonmatsu

**Affiliations:** 1 Theoretical Biology and Biophysics, Los Alamos National Laboratory, Los Alamos, NM 87545, United States; 2 New Mexico Consortium, Los Alamos, NM 87544, United States

## Abstract

Riboswitches are non-coding RNA sequences that control cellular processes through ligand binding. Conformational heterogeneity is fundamental to riboswitch functionality, yet this same attribute makes structural characterization of these mRNA elements challenging. Here, we use a combination of molecular dynamics and cryo-electron microscopy to expound the flexible nature of the glycine riboswitch tandem aptamers and characterize diMerent structural populations. We find that Mg^2+^ partially stabilizes the fully folded state, resulting in one-third of the particles adopting a unique “walking man” conformation, consisting of a rigidified core and two dynamic helices, and two-thirds adopting distinct, partially folded states. Glycine interactions double the relative population of fully folded particles by stabilizing a conserved inter-aptamer Hoogsteen base pair, enabling our capture of a 2.9 Å structure for this RNA-only system. The population data show that glycine and Mg^2+^ operate synergistically: glycine enhances Mg^2+^ occupancy, while Mg^2+^ drives glycine specificity. Our findings indicate that cryo-electron microscopy oMers a promising avenue to characterize RNA folding ensembles.

## Introduction

RNAs are structurally and functionally heterogeneous molecules that are essential and abundant in all known organisms. In bacteria, for example, RNA accounts for nearly 20% of the entire dry mass of the cell^1^. Despite their significance, RNA structure is still poorly characterized and diMicult to predict when compared to protein structure^2^. Indeed, of the >233,000 structures in the PDB in March of 2025, less than 1% are RNA-only structures, and a small fraction of those represent complex structures under 3 Å^3^. Issues with obtaining high-resolution structures of RNA are derived from the molecule’s two-fold heterogeneity. Firstly, the sugar-phosphate backbone has many degrees of freedom, resulting from seven torsion angles for each residue^4^. This leads to diMiculties in signal averaging at the atomic level. Secondly, RNA secondary and tertiary structures are surprisingly malleable and prone to kinetic trapping in local free energy minima^5^. Their folds are often highly dependent on solution conditions^6–8^ or minor diMerences in transcriptional start sites^9^. RNA structural characterization methods often produce either complex data resulting from the convolved signals of divergent states (as in the case of 2D probing methods or NMR), or a single snapshot of a dynamic system (as in the case of X-ray crystallography). While these methods are extraordinarily useful for describing some aspects of RNA structure, recent advancements in cryo-electron microscopy (cryoEM) data collection and processing have made it possible to simultaneously characterize heterogeneity and produce high-resolution information^10–13^, while removing signal from misfolded particles.

Riboswitches are RNA-based regulatory elements residing in mRNA that undergo structural changes in response to specific metabolites like glycine^14^, guanine^15^, SAM^16^, and many others. Structural heterogeneity is therefore fundamental to riboswitch functionality. Riboswitches are typically located in 5′-untranslated regions of mRNA, where they operate in *cis* to either transcriptionally or translationally regulate the expression of downstream genes^17,18^. Notably, riboswitches often control genes that metabolize or transport the very compounds that bind to them, creating an extremely eMicient feedback loop. Riboswitches generally consist of a ligand-binding aptamer domain, and a regulatory region, called the expression platform. Aptamer domain interactions with the ligand trigger global rearrangements in riboswitch structure, leading to either upregulation (ON-switch) or downregulation (OFF-switch) of target genes^18^. In some cases, such as in the glycine riboswitch family, homologous ON and OFF switches have both been identified, with downstream genes dictating the regulatory mechanism^19^.

**Fig. 1:**
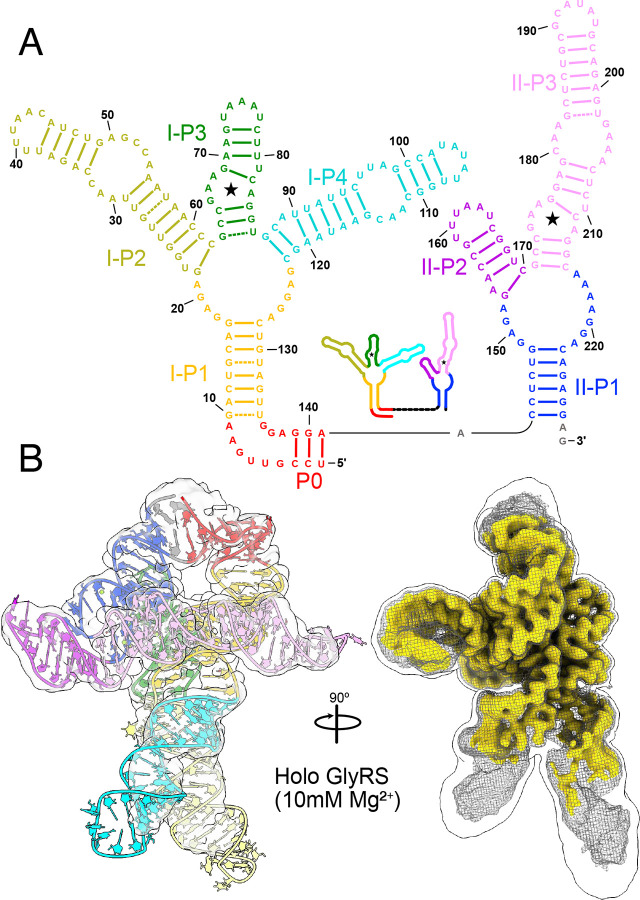
Overall walking man fold for the holo glycine riboswitch. **a** Secondary structural representation of the tandem glycine riboswitch aptamers, colored to highlight structural elements. Stars denote glycine binding pockets. A schematic representation is included as an inset. **b** Cryo-EM density of the holo glycine riboswitch structure, with the model colored consistent with *panel a*. A triple overlay of raw (mesh), sharpened (gold), and gaussian filtered (silhouette) maps is shown on the right to demonstrate how diMerent regions of the model were fit and refined.

Glycine riboswitches exhibit unusual functional and mechanistic diversity, extending beyond their simple ON/OFF role. DiMerent homologs can function as either transcriptional or translational riboswitches and may contain either a single glycine-binding aptamer domain or a pair of aptamer domains (Figure 1A). Riboswitches with two aptamer domains are referred to as “tandem riboswitches” and their functional states are often described using Boolean logic^20^. In the case of the glycine riboswitch, where both aptamers bind glycine molecules to regulate a shared expression platform, an AND logic gate is typically ascribed. This would mean that each aptamer must bind a glycine molecule to eMectively regulate the downstream gene.

The distinctive architecture of the tandem glycine riboswitch has long intrigued riboswitch researchers, leading to extensive biochemical and biophysical investigations^14,19,21–27^. Initial investigations demonstrated that some glycine riboswitch (glyRS) constructs displayed cooperative glycine-binding behavior, where binding of glycine to one aptamer increased the aMinity of the other aptamer for glycine^14,22,23^. However, subsequent research identified a conserved kink-turn interaction between a leader and linker region, which inhibits glycine binding cooperativity and facilitates both riboswitch folding and glycine interactions^28,29^. These findings led to the suggestion that the observed glycine binding cooperativity might have been due to the truncated constructs. The prevailing model posits that the function of the tandem glycine riboswitch involves a complex interplay between aptamer dimerization and glycine binding. Detailed analyses via equilibrium dialysis assays and site-directed mutagenesis indicate that a single aptamer is suMicient to drive glycine binding, and this interaction in turn stabilizes the tertiary structure of the riboswitch, facilitating gene regulation^21,26,27,30^. Importantly, RNA folds for riboswitches are also highly sensitive to the electrostatic environment of the solution due to their intricate tertiary folds and complex interactions with Mg^2+^ ions^31,32^. Cooperativity in riboswitches may be attributed to Mg^2+^ interactions leading to increased aMinity for target ligands^33,34^. Understanding how Mg^2+^ and other cations impact the populations of various conformational states of the ensemble is therefore imperative to understand RNA folding.

Here, we use a combination of cryo-EM and molecular dynamics simulations (MD) to characterize and depict the structural diversity inherent in the tandem aptamer domains of the *Vibrio cholerae* glycine riboswitch in three conditions: (i) without ligand or divalent cation, (ii) in the presence of Mg^2+^, and (iii) with both Mg^2+^ and glycine. Our analyses led to a “walking man” structure for the fully folded glycine riboswitch (Figure 1B), resolvable to 2.9 Å in the holo complex and to 3.3 Å in the absence of glycine. By exhaustively picking particles, we were able to generate a comparative ensemble of maps for each condition, which revealed several distinct core folds that are stable in the presence of Mg^2+^, and partially stabilized in the absence of Mg^2+^. While Mg^2+^ appears to shift the equilibrium toward folded conformations, and notably stabilizes the individual aptamers, glycine shifts the population of fully folded glycine riboswitch from approximately one to two thirds. Indeed, by comparing the diMerences in the electrostatic potential maps in the presence or absence of glycine, we show that a conserved inter-aptamer Hoogsteen base pair is held in a more consistent and proximal arrangement in the presence of glycine, leading to stabilization of the entire riboswitch fold.

Within the active sites, binding orientations for the glycines were fit based on the cryo-EM density and key interactions were supported by explicit solvent MD, providing a replicable method for combining mid-level resolution with MD to obtain biophysically reliable structures. Both glycine binding sites contain notable density for a coordinated Mg^2+^ that directly interacts with the carboxyl groups of the glycines. This Mg^2+^ density is either unidentifiable, or greatly reduced in the absence of glycine, supporting previous suggestions that there is heterotropic cooperative binding between Mg^2+^ and glycine^33,34^. Our data provide atomic-level details about the structure and dynamics of the glycine riboswitch, and demonstrate how key ligands modulate the stability of various populations within an RNA’s structural ensemble.

## Methods

### RNA Preparation

The *V. cholerae* gDNA template sequence used here (*GGCCTTCTAATACGACTCACTATAG*GTCCGTTGAAGACTGCAGGAGAGTGGTTGTTAACCAGATTTTAACATCTGAGCCAAATAACCCGCCGAAGAAGTAAATCTTTCAGGTGCATTATTCTTAGCCATATATTGGCAACGAATAAGCGAGGACTGTAGTTGGAGGAACCTCTGGAGAGAACCGTTTAATCGGTCGCCGAAGGAGCAAGCTCTGCGCATATGCAGAGTGAAACTCTCAGGCAAAAGGACAGAGGAG*TGAA*) includes both aptamer domains, but lacks the expression platform, and is based on the construct utilized in Kappel *et al*. (2020)^35^. The DNA template was generated by polymerase chain reaction using synthetic gblocks (IDT, Coralville, IA), with primers F: 5′ -GGCCTTCTAATACGACTCACTATAGG-3′ and R: 5′ -TTCACTCCTCTGTCCTTTTGCC-3′. The RNA was prepared through *in vitro* T7 RNA polymerase transcription using an AmpliScribe T7 High Yield Transcription Kit (LGC Biosearch Technologies; Hoddesdon, UK). The resulting RNA was then purified using RNAClean XP Beads (Beckman Coulter; Brea, CA) to the manufacturer’s instructions and eluted in sterile Milli-Q water. Five separate preparations of RNA were combined and concentrated using a vacuum concentrator to generate the final 2.5 mg/ml sample. RNA purity was verified using both native and denaturing gel electrophoresis.

### Cryo-EM sample preparation and data collection

To compare the impact of Mg^2+^ and glycine, three RNA samples containing final solutions with 1) no ligands or cofactors, 2) 10mM MgCl_2_, and 3) 10mM MgCl_2_ plus 2mM glycine were prepared in parallel and treated equally. Purified RNA was refolded using a modified version of the protocol described previously^36^. Briefly, RNA solutions containing 1.2mg/ml glycine riboswitch and 25mM MES (pH 6.0) were denatured at 95°C for 4 min and flash cooled on ice for 5 min. Either MgCl2, or MgCl_2_ and glycine were added to respective samples, and all three RNA solutions were then incubated at 37°C for 30 min. Samples were stored at 4°C until grids were prepared (approximately 1 hr).

Refolded RNAs were applied as 3.5 μl aliquots to Quantifoil R1.2/1.3 300-mesh gold grids (EM sciences, Prod. No. Q350AR1.3). Grids were glow discharged with a Gatan Solarus instrument for 30 s at 15 mA before sample application. Grids were blotted for 3 s in an FEI Vitrobot Mark IV (Thermo Fisher Scientific), set to 4 °C and 100% humidity, prior to plunge-freezing into liquid ethane.

Cryo-EM data were collected at the Columbia University Cryo-Electron Microscopy Center (CEC). Data were collected with a pixel size of 0.823 Å on a Titan Krios G3i (Thermo Fisher Scientific) operated at 300 kV using a Gatan K3 BioQuantum direct electron detector. A total of three Leginon^37^ data collections were used to generate the structures for the glycine riboswitch without MgCl_2_ or glycine, with 10mM MgCl_2_, and with 10mM MgCl_2_ and 2mM glycine. Data collection statistics are summarized in Extended Data Table 1.

### Cryo-EM data processing

**Extended Data Fig. 1:**
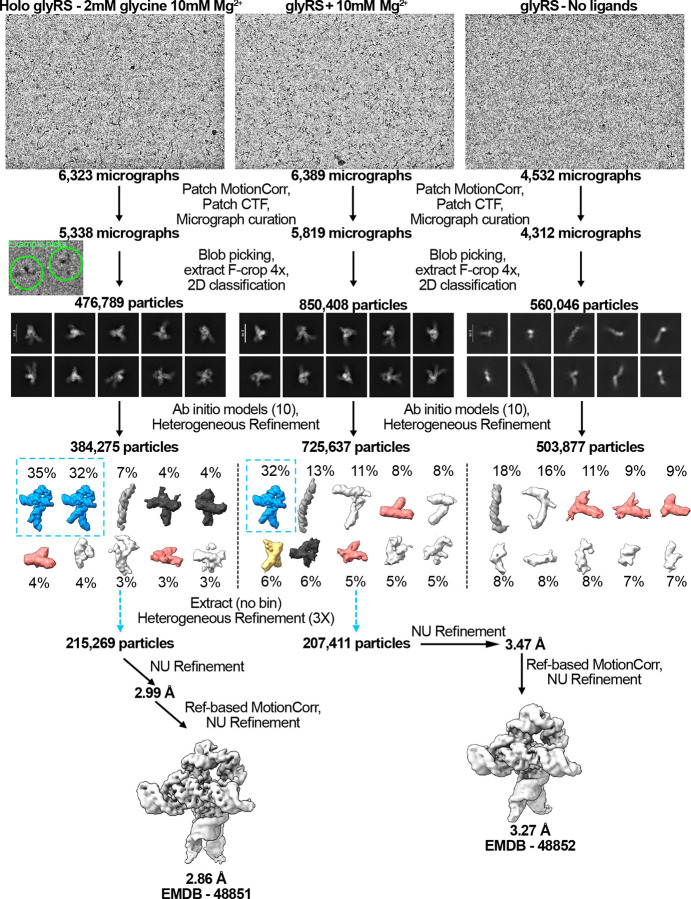
Cryo-EM data processing scheme. Data collection and processing procedures for the three glycine riboswitch datasets. From left to right: holo (2mM glycine, 10mM Mg^2+^), apo (no glycine, 10mM Mg^2+^), and neither glycine nor Mg^2+^.

The three datasets were processed using cryoSPARC (v4.6.0)^38^. The procedure is outlined in Extended Data Figure 1. Briefly, in all datasets, movie alignments, drift correction, and dose weighting were done using cryoSPARC’s patched implementations. Micrographs with poor CTF fits or non-ideal ice thickness were removed, resulting in 5338 micrographs for the holo dataset, 5819 micrographs for the MgCl_2_ dataset, and 4312 micrographs for the no-MgCl_2_, no-glycine dataset (Extended Data Table 1). For all datasets, particles were first auto-picked using cryoSPARC’s blob picker job to select particles with dimensions from 70Å to 120Å in an unbiased manner. Particles were then extracted with a box size of 256 px (210 Å) and datasets were cleaned using iterative 2D classification to remove obvious junk/spurious classes while conserving the heterogeneity in the sample. For each sample, 10 *ab initio* models were generated from cleaned particle pools to characterize how ligands and cofactors impact structural heterogeneity. Structures were then heterogeneously refined and aligned using cryoSPARC’s “Align 3D” job (Figure 3, Extended Data Figure 1)^38^.

To generate final maps for the MgCl_2_ and holo samples, 3D classes consistent with the fully folded construct were selected and filtered via successive rounds of 3D classification. Final datasets were obtained via reference-based motion correction, and non-uniformly refined^39^ without adaptive marginalization or dynamic masking to produce the final maps. Map resolution was determined by the Fourier shell correlation (FSC) between two half-maps at a value of 0.143 with FSC-mask auto-tightening, resulting in ostensible resolutions of 2.9 Å (215,269 particles) for the holo sample, and 3.3 Å for the MgCl_2_ sample. To better understand the dynamic motions present in the holo complex, the final particle pool was 3D-classified into four maps at a filter resolution of 5 Å (Figure 4). Structural heterogeneity was further explored via 3D-variability analysis with 20 models at filter resolutions of 5 Å (Movie 1 and Movie 2)^40^. Although attempts were made to further refine and resolve the leg regions using various cryo-EM toolkits that focus on dynamic regions, we were unable to obtain a high-resolution reconstruction of the leg regions.

### Model building and refinement

**Extended Data Fig. 2:**
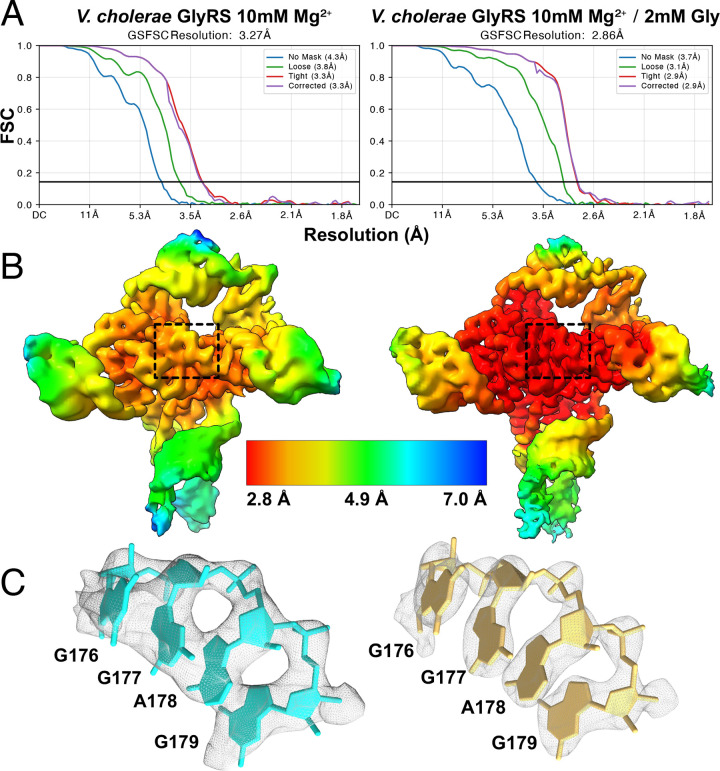
Overall and local resolution estimation. **a** Fourier Shell Correlation (FSC) plot generate in cryoSPARC for the holo (left) and apo (right) glycine riboswitch structures. **b** Surface representations of the respective maps colored based on local resolution. **c** Expanded view of the boxed region in *b*, showing the density and model fit in the core region.

A low-resolution structure of the *V. cholerae* glycine riboswitch is available (PDB-6WLT)^35^, which was used as the initial template for modelling our structures in Coot (v.9.8.95)^41^. RNA A-form restraints were used extensively to ensure low-resolution areas did not collapse during refinement. Model geometries and fits to maps were adjusted in ISOLDE (v1.8)^42^ to decrease issues arising from steric clashes, with restraints placed on RNA based pairs. Low-resolution regions in the “legs” and “left arm” were refined in a filtered map to ensure backbone placement was reasonable, but care should be taken to interpret finer details in any of these low resolution regions (Extended Data Figure 2). As it is diMicult to distinguish well-occupied Mg^2+^ sites from water in cryo-EM electrostatic potential maps, decisions on which molecule to model were based on either biochemical evidence (such as within the glycine binding site), or derived from homologous regions of a previously available 3.55 Å crystal structure of the related *Fusobacterium nucleatum* glycine riboswitch (PDB-3P49)^23^. Where neither method was applicable, ligands were left unmodelled to avoid overinterpreting the data. Final refinements were performed using PHENIX (v1.21.1-5286-000)^43^ real space refinement against the final maps for both the holo and MgCl_2_ samples. Model statistics are disclosed in Extended Data Table 1. Structures and maps were visualized and presented using ChimeraX^44^.

### Explicit solvent molecular dynamics simulations

The structure of the glycine riboswitch obtained in the 10 mM MgCl_2_ plus 2 mM glycine condition was used to perform all-atom explicit solvent molecular dynamics simulations. The riboswitch structure was solvated with the TIP3P water molecules and 10 mM MgCl_2_. Notably, a bulk concentration of 10 mM Mg^2+^ ions (bulk is defined by the region >12 Å away from the RNA) was achieved following conducting several cycles of equilibration stages using the protocol described previously^45^. Moreover, 140 mM K^+^ ions were added for neutralizing the whole system to net charge zero, which resulted in bulk concentration of 80 mM K^+^. The resulting systems had approximately 240,000 atoms in total. The amber M99bsc0_χOL3_ force field for RNA^46^, amber ff19SB for zwitterionic glycine^47^, and recently optimized parameters for the ions Mg^2+^, K^+^, and Cl^−^ with TIP3P water were used^48,49^. The cut-off for short-range electrostatics and the Van der Waals interactions was 12 Å, and the PME method^50^ was used for the long-range electrostatics with a grid size of 1.2 Å. Hydrogen-containing bonds were constrained using the LINCS algorithm to enable a timestep of 2 fs^51^. To check the stability of glycines in Apt-I and Apt-II in our proposed orientations, we used four unbiased simulations for the timescale of 1 μs. All MD simulations were carried out using the GROMACS v2024.3^52^ patched with PLUMED v2.9.2^53^.

### Metadynamics explicit solvent molecular dynamics simulations

To further support our proposed glycine orientations, we employed the combined well-tempered and multiple walker metadynamics technique^54,55^. To enforce reorientation of the glycine in both aptamers, two collective variables for Apt-I and Apt-II defined as the angle between the *z*-components of vector passing from amino nitrogen to carboxyl carbon atoms of a glycine molecule and *z*-axis^56^. To define these collective variables, the whole riboswitch was aligned with respect to *z*-axis as shown in Figure 6A, and the phosphorus atom position was restrained during the simulations. Three independent simulations for a total timescale of 2 μs were carried out. In each simulation two metadynamics runs, each having 8 walkers, were performed simultaneously by biasing the angle collective variables defined for Apt-I and Apt-II^57^. Along the collective variable, Gaussians with the height of 1 kJ/mol and width of 1° were deposited at the interval of 2 ps. The bias factor was set to 15, corresponding to tuning temperature of 4200 K. Upon completion of simulations, two-dimensional free energy surfaces as a function of collective variables, distance between Mg^2+^ and center of mass of glycine and the angle of glycine with respect to z-axis, were calculated using the Tiwary-Parrinello reweighting scheme^58^.

### Flexible simulations using SMOG

The initial cryo-EM structure was used to generate the SMOG native contact potential^59^. All simulations were performed using the MD software package GROMACS^60^. The initial configuration and topology files for GROMACS were generated using the SMOG web tool^61^. The starting structure was minimized for 10,000 steps using the SMOG native contact potential. MDFIT^62,63^ and cryo_fit^64^ were employed for flexible fitting into the generated cryoEM map. To accurately match the cryo-EM density map with the configurational map, a 10^5^-step MD simulation was conducted to evolve the simulation towards a stable structure. Principal component analysis (PCA) was performed on the MD trajectories to gain insights into collective motion. The inbuilt subroutine, gmx covar, in GROMACS was used to generate both eigenvalues and eigenvectors, followed by filtering the individual eigenvectors to analyze the two largest eigenvalues. For visualization, VMD^65,66^ and UCSF Chimera were utilized.

## Results

### Holo state of the glycine riboswitch adopts ‘walking man’ fold

To better categorize populations within the folding ensemble, it is important to have a well-resolved reference structure to align partially folded arrangements. Previous small-angle X-ray scattering studies by Lipfert *et al*. (2007, 2010) have produced low-resolution descriptions of the tandem glycine riboswitch, which demonstrated that it is most stable and compacted in the presence of both Mg^2+^ and glycine^25,34^. We therefore first sought to characterize the *V. cholerae* glycine riboswitch in a solution containing 10mM Mg^2+^ and 2mM glycine to obtain a reasonably homogeneous sample. Under these conditions, we were able to generate a 2.9 Å electrostatic potential map for the holo glycine riboswitch, which folds into a conformation resembling a “walking man” (Figure 1B, SI Figure 1). The core torso region of this fold (Aptamer-I P1 and P3; Aptamer-II P1 and P3) is well resolved, with clear density around base pairs. In contrast, the arm (Aptamer-II P2 and P3) and leg regions (Aptamer-I P2 and P4) are not as well resolved (Figure 1B, Extended Data Figure 2), despite utilizing several methods that attempt to characterize and refine dynamic regions in cryo-EM structures. This may be due to the relatively small size and multidirectional stepwise motion of the leg regions (described below).

Although it can be diMicult to build initial models for RNA-only systems without collecting 2D probing data^35,67^, prior work on a holo system for a diMerent species (*F. nucleatum*), which did not explore the conformational ensemble, yielded a 3.55 Å crystal structure of the glycine riboswitch stabilized by a proteinaceous partner. An excellent previous study also used cryoEM to obtain a 5.7 Å structure of the holo *V. cholerae* glycine riboswitch, which is 35% longer than the *F. nucleatum* homolog^23,35^. While 5.7 Å is insuMicient resolution to make out finer molecular details, this structure, in combination with the *F. nucleatum* X-ray crystallography structure, served as excellent starting points to build our model (Figure 1B).

As it is diMicult to distinguish well-occupied Mg^2+^ sites from water in cryo-EM electrostatic potential maps, decisions on which molecule to model were based on either biochemical evidence (such as within the glycine binding site), or derived from homologous regions of a previously available 3.55 Å crystal structure of the related *Fusobacterium nucleatum* glycine riboswitch (PDB-3P49)^23^. X-ray crystallography can also take advantage of electron counts, occupancy, and coordination to attempt to distinguish Mg^2+^ from water^68,69^. The homologous *F. nucleatum* crystal structures of the full length^23^ and aptamer constructs^24^ were therefore also utilized to corroborate solvent and ion assignments when biophysical evidence was insuMicient. Where neither method was applicable, ligands were left unmodelled to avoid overinterpreting the data.

**Extended Data Fig. 3:**
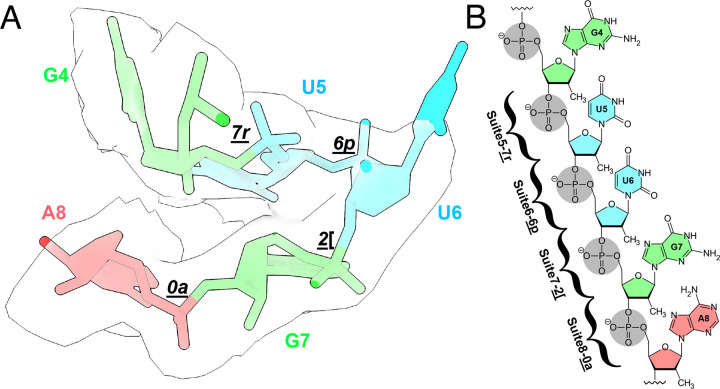
The P0 region of the glycine riboswitch adopts RNA suites consistent with a kink-turn conformation. **a** Overlay of residues 4–8 in the holo glycine riboswitch structure with a transparent density map. Colors correspond to base identity. Suites for each sugar-to-sugar set of torsion angles are included. **b** Chemical view of each nucleotide and suite in the kink-turn.

**Extended Data Fig. 4:**
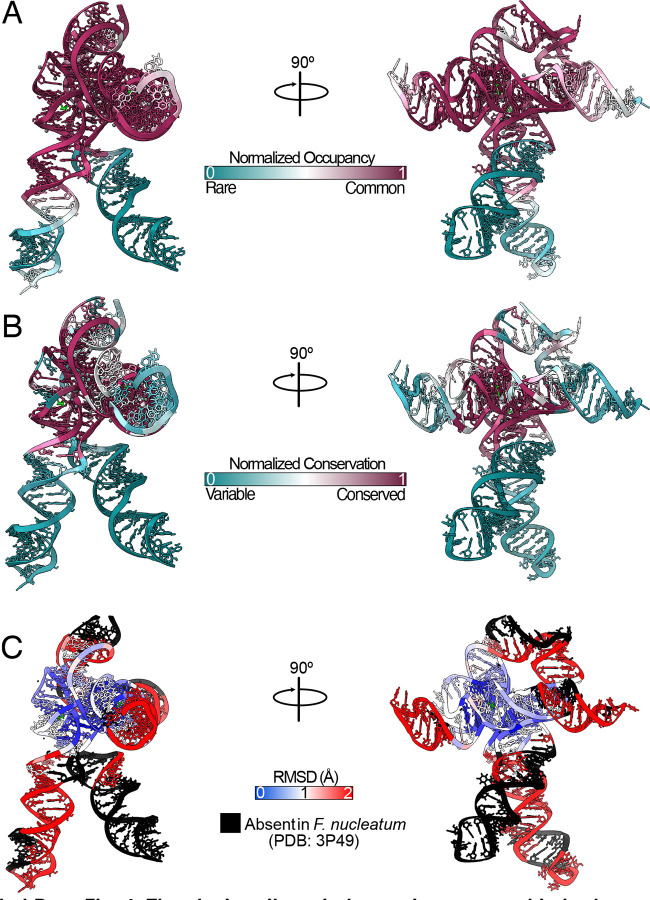
The glycine riboswitch core is conserved in both sequence and structure. **a** Normalized occupancy of each residue within the *V. cholerae* glycine riboswitch across all identified homologs. **b** Normalized conservation for each residue within the *V. cholerae* glycine riboswitch across all identified homologs. Normalized values were calculated with gaps included. Values from *a,b* were calculated using 11,626 glycine riboswitch sequences identified, and alignments made, in Torgerson *et al*. (2020)^21^**. c** RMSD calculated in ChimeraX^44^ using the *F. nucleatum* glycine riboswitch (PDB code: 3P49)^23^ and the *V. cholerae* structure solved here. Regions colored in black are absent in *F. nucleatum*.

**Fig. 2:**
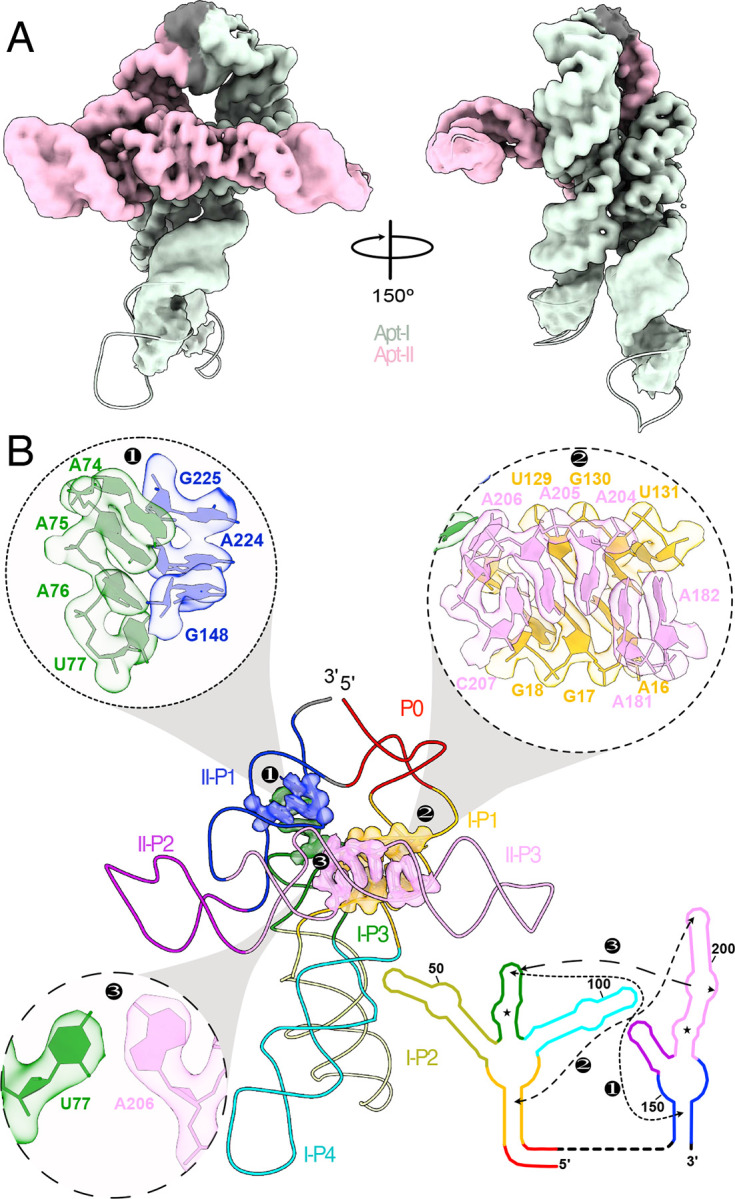
The full glycine riboswitch fold is stabilized by three main regions of inter-aptamer contacts. **a** CryoEM map and model colored to highlight residues derived from Apt-I (light green), Apt-II (pink), or linker regions (grey). **b** Regions of Inter-aptamer contact. Residues of one aptamer that approach within 2.5 Å of the other aptamer were considered as a contact, leading to three regions involved in stabilization of the fully folded conformation. This includes two sets of A-minor motifs (*1,2*), and one Hoogsteen base pair (*3*). CryoEM density for contact regions is extracted to demonstrate fit. A schematic representation of these contact regions is included (right) to emphasize regions involved in stabilization of the full fold. Model and schematic representation are colored to emphasize structural elements.

Our cryo-EM study shows that the tandem aptamers of the *V. cholerae* glycine riboswitch adopt distinct folds, with aptamer one (Apt-I) forming a 4-way junction to fashion the back and leg helices, while aptamer two (Apt-II) forms a 3-way junction to create the arms and head region (Figure 1, Figure 2). The global fold is stabilized by three main inter-aptamer interaction zones: 1) a conserved Hoogsteen base pair (U77-A206 here) between the P3 regions^23^, 2) three adenines in the loop of Apt-I P3 buried in the minor groove of Apt-II P1, and 3) the reciprocal interaction with four adenine bases of Apt-II P3 binding to a loop in Apt-I P1 (Figure 2). Our findings are consistent with previous chemical probing and crystallography work, which identified these “A-minor” motifs and demonstrated that P1 and P3 are conserved, while P2 and P4 are variable^21–24,27^. The kink-turn motif is positioned to contribute to tandem aptamer stabilization. We visualize this region and confirm that nucleotides G4-A8 adopt a conformation consistent with a kink-turn motif (**7r6p2[0a**^70^; Extended Data Figure 3). Interestingly, the regions of the *V. cholerae* glycine riboswitch that are best resolved (Figure 1B, Extended Data Figure 2) mirror those that are structurally most conserved among the *V. cholerae* and *F. nucleatum* glycine riboswitches (Extended Data Figure 4). These regions also display the highest level of sequence conservation across all known homologs (Extended Data Figure 4)^21^. This indicates that these folds represent the “core” glycine riboswitch and may provide a means to develop a minimized functional version of this regulatory RNA.

### Exploring the conformational ensemble by investigating non-holo states: Glycine remodels the conformational landscape, stabilizing the fully folded conformation

**Fig. 3:**
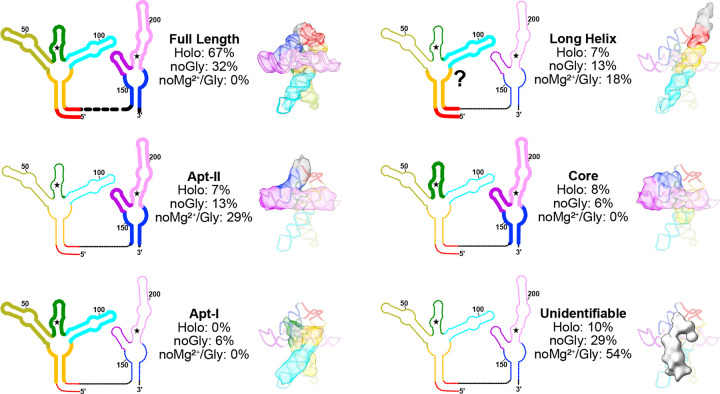
Populations of glycine riboswitch folds are highly responsive to solution conditions. Six general classes of structures are show as both a schematic representation and with a representative transparent map overlaid with a potential structural element from the model. Fractional populations within the holo, no glycine, and no glycine/Mg^2+^ sample are noted for each class. A question mark is used to emphasize the uncertainty inherent in defining which specific residues are involved in the structure.

To understand how glycine and Mg^2+^ aMect the ensemble of configurational populations of the glycine riboswitch, we broadly picked particles from cryo-EM grids in three conditions: 1) holo glycine riboswitch with 10mM Mg^2+^ and 2mM glycine, 2) glycine riboswitch with 10 mM Mg^2+^ without glycine, and 3) glycine riboswitch without Mg^2+^ and without glycine. Particles were used to generate ten *ab initio* models for each condition, which were then heterogeneously refined into ten representative structures of the conformational ensemble. When glycine and magnesium are both present, approximately two thirds% of all particles are fully folded (Figure 3 and Extended Data Figure 1). In addition, several distinct classes that resemble the core of the riboswitch are identifiable, as well as one fold reminiscent of Apt-II alone (Figure 3 and Extended Data Figure 1). Our results are consistent with those of previous studies that show that Apt-II is the driver for glycine binding in *V. cholerae* and the structurally more stable aptamer^21,24,27^.

The population of fully folded RNA is significantly lower when glycine is absent (Figure 3 and Extended Data Figure 1), accounting for only one third of all picked particles. Instead, the relative population of the potential Apt-II fold is doubled, and a new conformation consistent with an Apt-I fold is now identifiable (Figure 3 and Extended Data Figure 1). The presence of a possible Apt-I class in the absence of glycine, as well as the strong enrichment of fully folded particles when both ligands are present, suggests that glycine helps to stabilize inter-aptamer contacts to shift the single aptamer populations towards the fully folded state.

Nearly 30% of particles belong to unidentifiable classes in the Mg^2+^-alone sample, compared to 10% in the holo sample (Figure 3 and Extended Data Figure 1). While these low-resolution structures could be artifacts evolving from insuMicient statistical coverage, the shift of populations upon addition of ligands suggests that they are instead intermediates or oM-target, misfolded conformations. In some cases, similar but unidentifiable folds are present in both samples containing Mg^2+^ (Extended Data Figure 1). It may therefore be possible to use cryo-EM to characterize potentially relevant conformations in RNA folding pathways.

### Synergy between Mg^2+^ and glycine: the glycine riboswitch requires Mg^2+^ to adopt the fully folded conformation

In contrast to the cases discussed above, no fully folded particles were discernable in the absence of both Mg^2+^ and glycine (Extended Data Figure 1, Figure 3). Nearly 30% of all picked particles instead adopted a “7-shaped” fold, akin to a portion of the Apt-II structure (Figure 3, Extended Data Figure 1). The relatively lower resolution of maps from these classes indicates that the RNA is dynamic, leading to poor signal averaging. Interestingly, long RNA helices were identifiable in all three conditions, with the population decreasing as the fully folded population increased (Figure 3). These data suggest that either a single long helix, such as the one encompassing P1 and P2 of Apt-I, is particularly stable, or that RNA chains form helical assemblies in solution to facilitate burial of bases.

### The glycine riboswitch is highly heterogeneous in the leg regions when fully folded

**Fig. 4:**
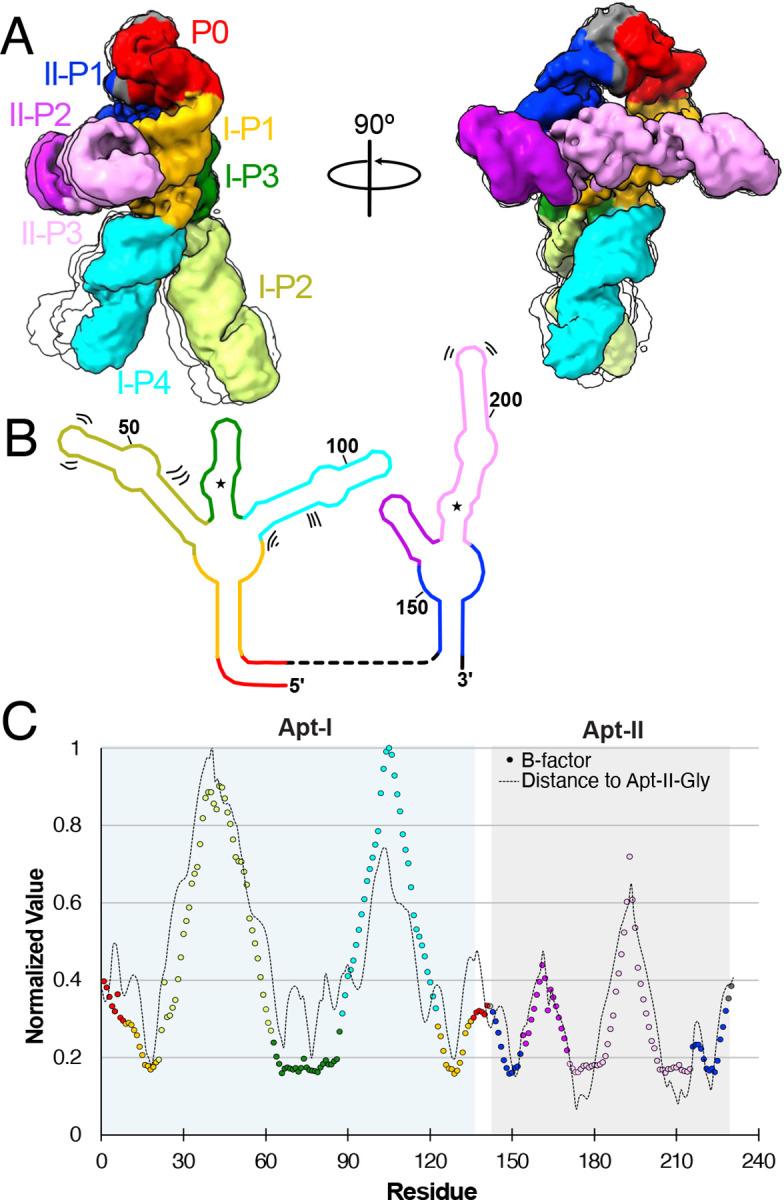
The glycine riboswitch P2 and P4 regions are highly dynamic. **a** Four representative models derived from 3D classification of the final holo particle pool, limited to 5 Å resolution. One structure is colored based on structural elements, while three others are shown as simple transparent silhouettes. **b** Schematic representation of secondary structure, with bending and shifting motions denoted by sets of two or three lines. **c** Normalized B-factor (colored circles) and distance of Apt-II glycine (dashed line) per residue for the holo glycine riboswitch cryoEM structure. Circles are colored consistent with structural elements in *a,b*. Apt-I and Apt-II regions are shaded in blue and grey, respectively.

The degrees of freedom in the RNA backbone manifest not only an ensemble of folds, but also dynamic regions within the folds. To characterize glycine riboswitch dynamics and identify potential hinge regions, we used two approaches: (i) a 3D classification and 3D variability analysis on our final holo particle pool (Movie 1, Movie 2, Figure 4)^40^, and (ii) structure-based molecular simulations. With the first method, we identified three distinct movements within the *V. cholerae* glycine riboswitch. First, we observe that the distance between the feet regions increases from 51 Å to 61 Å, and their tangent-tangent angle increases from 51° to 81°. The overall motion the of the leg regions resembles a stepping motion (Movie 1). Second, the core hip regions flex simultaneously, leading to the legs pivoting forward by approximately 9° (Movie 2). The final motion visible is within the head region, where our low-resolution analysis catches movement in the 3’ and 5’ ends of the RNA (Movie1, Movie 2). These movements are partially recapitulated in the PHENIX-derived B-factors, which show that the legs are the most dynamic region (Figure 4). Interestingly, the normalized B-factors correlate well with the distance between each nucleotide and the Apt-II glycine. The glycine binding site is therefore likely to be the most stable part of the holo riboswitch. Although it is unclear what role the more dynamic regions play in riboswitch functionality, it is of note that the most dynamic portion (i.e. Apt-I P4) is present in only 13% of glycine riboswitches^21^.

To further evaluate the dynamic motions obtained via cryo-EM, we used all-atom structure-based molecular simulations. The RMSF values mirrored the PHENIX-derived B-factors, and the two distinct stepping motions seem in cryoEM were mirrored in the principal component analysis motions derived from the molecular simulations (Movie 3, Movie 4), indicating that molecular simulations can capture molecular motions similar to those derived from cryo-EM variability analysis.

### Glycine and Mg^2+^ bind in a synergistic manner and stabilize inter-aptamer contacts

**Fig. 5:**
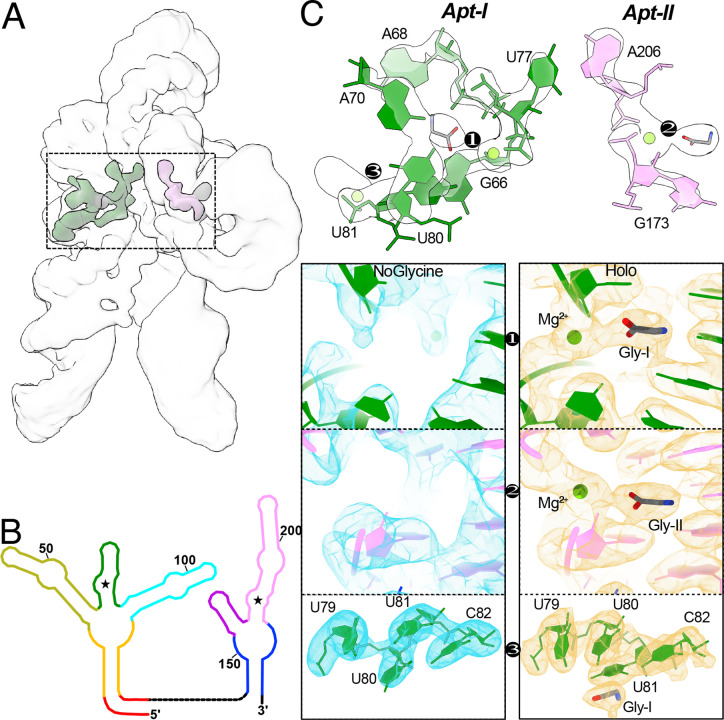
Glycine and magnesium synergistically stabilize inter-aptamer glycine riboswitch contacts. **a** Transparent cryoEM map of the holo glycine riboswitch, overlaid with a gaussian filtered diMerence map of the holo glycine riboswitch minus the apo glycine riboswitch, demonstrating structural changes are localized to the glycine binding loci. **b** Schematic representation of the glycine riboswitch secondary structure, colored to emphasize diMerent structural elements. **c** Enlarged view of regions altered in the presence of glycine, with residues colored consistent with *panel b* and overlaid with a silhouette view of the diMerence map. Glycine ligands are colored in grey and magnesium ions are shown in lime green. Note the direct path of stabilization between the two glycine residues, connected via the conserved inter-aptamer Hoogsteen base pair (here, U77-A206). Three key regions are further enlarged, with modified density in the Apt-I (*1*), and Apt-II (*2*) binding pockets, demonstrating a cooperative interaction between magnesium and glycine in these regions. Movement of U80 and U81 (*3*) may capture movement required to allow glycine access to the tight binding pocket.

The conformational ensemble analysis demonstrated that glycine stabilizes the fully folded conformation. To understand the mechanism of stabilization, we solved the glycine riboswitch structure for the most enriched class in the presence of 10 mM Mg^2+^ and compared it to the holo structure. Notably, despite using the same RNA preparation and a similar number of particles (216K in the holo sample and 207K with Mg^2+^ alone), the resolution was significantly lower in the absence of glycine, yielding a 3.3 Å structure. This diMerence (*i.e*., lower resolution in the absence of glycine vs. higher resolution in the presence of glycine) may be indicative of structural rigidification by the bound glycine. The overall fold for our RNA construct, which lacks the regulatory expression platform, is essentially identical under the two conditions (Extended Data Figure 1). Indeed, a diMerence map of the electrostatic potentials under either condition demonstrates that all the major changes take place in a localized region surrounding the glycine binding sites (Figure 5).

As depicted in the RNA secondary structure diagram (Figure 1A), the two glycine binding sites (denoted by star symbols) reside on diMerent aptamers and appear to be quite distant from each other. Interestingly, in 3D, a clear interaction path is visible between the two glycine binding sites, as seen in the diMerence map (Figure 5C). From Apt-II, glycine binding leads to increased density for an adjacent Mg^2+^ connected to A206. This adenosine forms the conserved Hoogsteen base pair to U77 in Apt-I^23^, which is also stabilized by an attached Mg^2+^ bound to the Apt-I glycine. These data evince a role for glycine where binding leads to stabilization of the fully folded tandem aptamer by rigidifying the inter-aptamer Hoogsteen base pair. It is notable that the magnesium density within the glycine binding pocket is either absent (as in Apt-II) or significantly diminished (as in Apt-I) in the absence of glycine (Figure 5C), even though the magnesium concentration is held constant. Previous work has demonstrated that metabolites and cations bind cooperatively to riboswitches, where aMinity is increased for the metabolite when cations are available, and vice-versa^33^. While many studies describe cations as working as an ion cloud to oMset the phosphate charges in the RNA backbone, our data reveal that the glycine riboswitch also has two specific Mg^2+^ binding sites that synergistically interact with glycine within the binding pockets.

**Extended Data Fig. 5:**
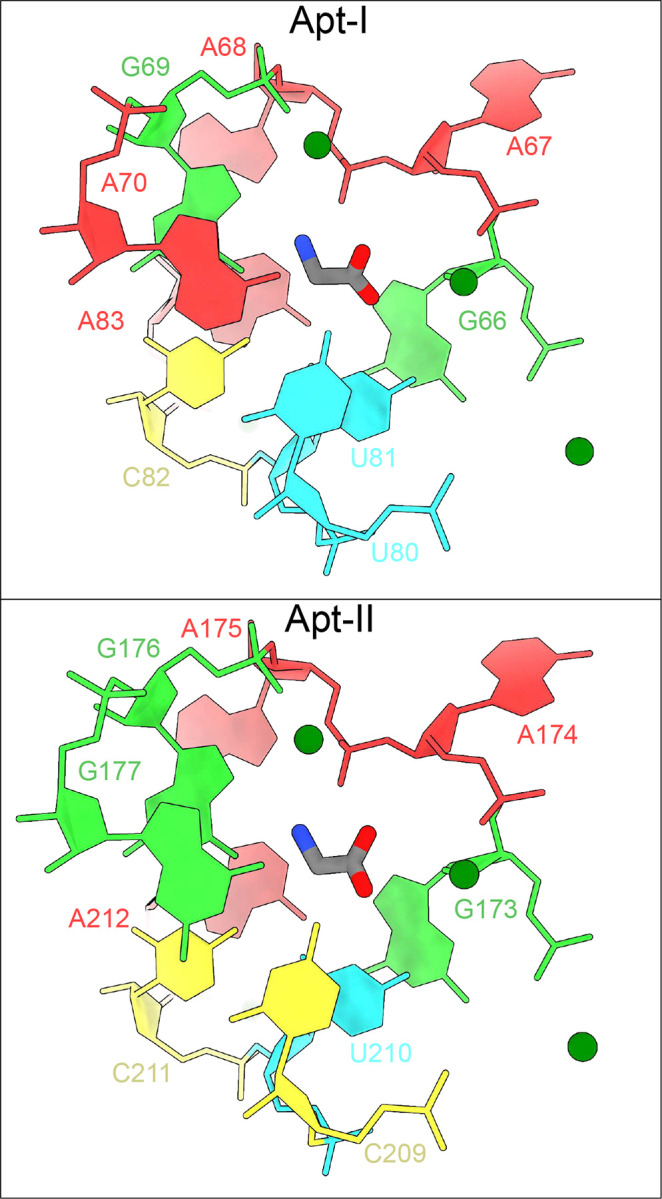
Apt-II is stabilized by an additional G-C pair proximal to the glycine binding site. Residues within 4 Å of the bound glycine are shown for both Apt-I (top) and Apt-II (bottom). Glycines are colored with carbons in grey and magnesium ions proximal to the binding site are shown and colored dark green. Nucleotides are colored according to base identity. Binding sites are largely identical, with the exception of the A70-U80 (Apt-I) to G177-C209 (Apt-II) change.

Crystallographic studies on individual glycine riboswitch aptamers indicate that glycine fits very snugly within the binding pocket and that the glycine itself is trapped by adjacent magnesium ions^24^. Our data similarly show compact binding pockets surrounded by several Mg^2+^ (Extended Data Figure 5). In the Apt-I pocket, U80 and U81 undergo significant conformational transitions between the bound and unbound states (Figure 5C, Movie 3). If the nucleotide U80 were in its apo conformation, it would directly clash with bound glycine, thus necessitating this conformational shift. Additionally, the poorer density in this region in the absence of glycine suggests that these residues are more dynamic in the apo state. This conformational transition may therefore represent a potential pathway for glycine access to the binding pocket.

### Explicit solvent molecular dynamics simulations help validate glycine orientations

Previous crystallography studies on the *F. nucleatum* glycine riboswitch at 3.55 Å concluded that the Apt-II glycine is stabilized by direct interactions between the carboxyl group and the equivalent of U81, as well the amino group and G69^24^. Although the 2.9 Å structure generated here is of insuMicient resolution to confidently orient a small glycine ligand, our data are more consistent with an alternate arrangement where the nitrogen group points towards the adjacent phosphate from residue A68 (Extended Data Fig. 5). To distinguish between these two potential conformations, we performed explicit solvent all-atom molecular dynamics simulations using particle mesh Ewald electrostatics, with an environment of explicit bulk magnesium and potassium ions at 10 mM MgCl_2_ and 80 mM K^+^.

**Extended Data Fig. 6:**
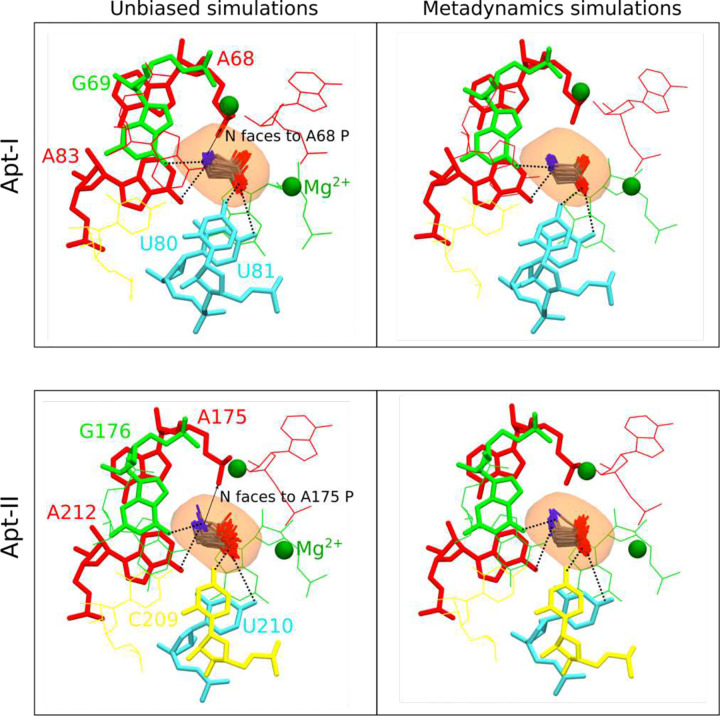
Glycines remain stable in our proposed configurations for Apt-I and Apt-II. From unbiased and metadynamics simulations trajectories, several thousand frames representing the initial state conformations (Fig. 6) are extracted, and approximately one hundred conformations are depicted for glycine in each aptamer. Glycines maintain constant distance from the Mg^2+^ ions, with the carboxyl group facing to towards the Mg^+2^ ions and the amino group orientated towards the phosphate group of A68 and A175 in Apt-I and Apt-II, respectively, indicating our proposed glycine configurations are most stable.

**Extended Data Fig. 7:**
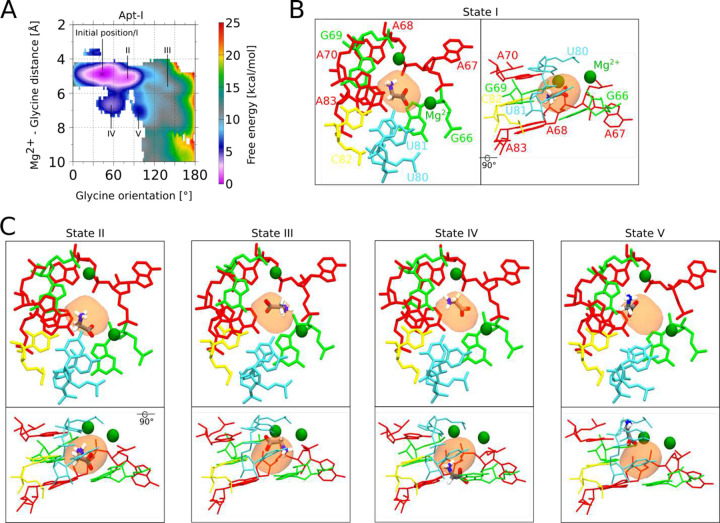
Glycine conformations in metastable states seen in vicinity of modeled state. **a** Free energy surfaces with respect to the Mg^2+^-glycine distance and glycine orientation for Apt-I (consistent with Fig. 6b). Five minima over the free energy surface, including a minimum representing the initial state of glycine modeled from the cryo-EM map, denoted as state I, are marked. **b** Conformation of glycine in minimum I, along with the neighboring residues, Mg^2+^ ions and cryo-EM-derived, low-pass filtered glycine density, are shown from two perspectives. **c** Similarly, glycine conformations in four additional minima are illustrated.

**Fig. 6:**
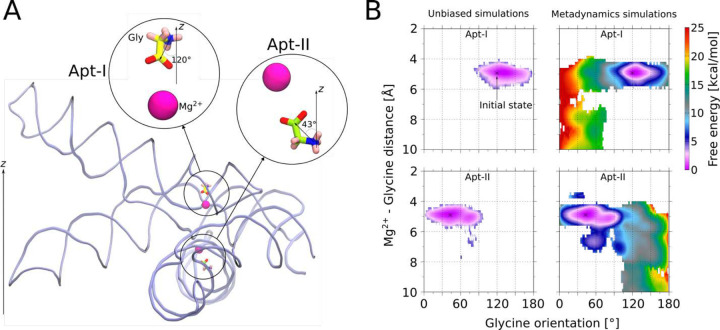
Glycine binding orientation analysis using all-atom explicit solvent MD simulations. **a** Glycine orientation is defined as the angle between the *z*-components of the vector passing from amino N to carboxyl C of glycine and the *z*-axis. To facilitate the angles calculations, the whole riboswitch structure is aligned to *z*-axis in the same manner as shown in the figure. Initial angles for Apt-I and Apt-II glycines are mentioned on subset panels. **b** Free energy surfaces with respect to two variables: i) distance between the Mg^2+^ and the center of mass of glycine, and ii) glycine orientation as described in *panel a*. Surfaces on both left panels for Apt-I and Apt-II glycines are obtained by running four 1 μs long equilibrium (unbiased) simulations, while on the right are derived after performing three 2 μs long metadynamics simulations, where the above described angle variables for both glycines were sampled to reorient glycines in all possible orientations. The starting position of the glycines with respect to both variables are marked with the * symbol on all panels.

First, using the unbiased simulations, we confirm that both glycines maintain our proposed orientations with carboxyl groups facing toward with Mg^2+^ ions, and with glycine-Mg^2+^ distances/angles conserved (Figure 6B). In this position and orientation, the amino group of both glycines are consistently directed towards the phosphate of A68 in Apt-I and A175 in Apt-II (Extended Data Fig. 6). Subsequently, enhanced-sampling explicit solvent metadynamics molecular dynamics simulations were carried out that sample the glycine molecules at all orientations. The resulting free energy surfaces show that when glycine maintains the 4 to 6 Å distance from Mg^2+^ ions, our proposed configuration yielded the most favorable energy basins for both aptamers. Inside these basins, the glycine amino groups always remained oriented towards the phosphate of A68 and A175 in respective aptamers (Extended Data Fig. 6), consistent with our proposed cryo-EM structure. Notably, four other potential metastable states seen for Apt-I pull the displaced glycine out of the corresponding experimental density (Extended Data Fig. 7). Both the explicit solvent molecular dynamics simulations and explicit solvent molecular dynamics simulations with metadynamics help to corroborate our proposed glycine orientations. These data also suggest that glycines bind to *V. cholerae* glycine riboswitch in an orientation that diMers from previously described systems. This workflow provides a means to combine mid-resolution cryoEM data with MD to provide higher confidence about ligand-complex orientations.

## Discussion

Riboswitches play an important role in metabolism and regulate approximately 4% of bacterial genes^71,72^. Riboswitch function is often described as regulation via alternation between two mutually exclusive conformations; however, this description belies the ensemble nature of biological macromolecules. In proteins, for example, conformational ensembles can be used to describe the mechanism of action for an enzyme or provide information about the biological role of disordered regions^73,74^. For RNA, an ensemble description is even more germane, as the significant flexibility inherent to the RNA backbone allows for the adoption of a greater variety of conformations^4,64,75,76^. NMR studies on the 2’ deoxyguanosine riboswitch have demonstrated that the RNA forms a multifarious set of ON, OFF, and intermediate conformations, even in the presence of ligand^77,78^. In fact, in vitro transcription experiments suggest that only 70% of the particles in some systems adopt a ligand-bound conformation in the presence of metabolite^77^. Riboswitch operation can thus be described by population states within an ensemble that shift in accordance with changing buMer conditions^79^. Here, cryoEM explicitly captures this shift in populations and may, in future studies, illuminate the RNA folding pathway via characterization of low population states.

Our study shows that the glycine riboswitch adopts a highly dynamic “walking man” conformation, with several distinct stepping motions that coalesce in a well-resolved core surrounded by flexible loops and helices (Figure 1, Figure 4). The lack of conservation in the dynamic regions (Extended Data Figure 4)^21^ suggests that their role is not sequence specific. One potential explanation for this is that they encode protein binding sites, as has been seen in the *F. nucleatum* glycine riboswitch^23^, that vary dramatically between species. Alternatively, many studies have described a kinetic attribute in riboswitch regulation, where transcriptional pause sites provide time for the RNA structural rearrangements to mediate gene expression^77,78,80^. It is therefore possible that these flexible regions modulate the eMect of ligand binding on expression levels by serving as an alternative to transcriptional pause sites.

Within each binding pocket, a coordinated Mg^2+^ is proximal to the bound glycine (Figure 5). The marked reduction of magnesium density in the absence of glycine is notable. While this could indicate that this region is flexible in the absence of glycine, the local resolution of adjacent residues of this core region (Extended Data Figure 2) suggests this region is relatively restricted. The data are instead more consistent with the absence or low occupancy of magnesium at these loci in this case. We therefore hypothesize that glycine stabilizes Mg^2+^ within the binding pocket, and that this interaction is one of the main drivers for glycine riboswitch specificity. In support of our conclusion, prior studies revealed a reciprocal cooperativity between ligands and Mg^2+^, with the presence of one binder increasing the aMinity of the other^33^. Additionally, one prescient study used SAXS data (radius of gyration) to suggest that there are two specific divalent ion binding sites in the *V. cholerae* glycine riboswitch, which interact cooperatively with glycine to result in the final compact structure^34^. This series of RNA-ligand-cation interactions strongly stabilizes the core fold of the glycine riboswitch, suggesting an explanation for the larger question of why this riboswitch uses two adjacent aptamer domains. Based on our data, the presence of two aptamer domains helps the riboswitch achieve a more stable or robust structure. The linked domains may also provide structural redundancy, ensuring that the riboswitch folds correctly and functions eMiciently across diMerent environmental conditions.

Previous work on the *V. cholerae* glycine riboswitch has demonstrated that Apt-II binds glycine more tightly than Apt-I^24^. In addition, glycine binding by Apt-II is an enthalpically-driven reaction, while Apt-I interactions are entropically driven^24^, despite having nearly identical binding sites (Extended Data Figure 5). These results suggest that more distal factors, such as the overall fold of the aptamer, influence the thermodynamics of glycine-aptamer interactions. Our ensemble analysis indicates that Apt-II is more stable than Apt-I in *V. cholerae* (Figure 3), potentially resulting from the extra G-C bond present within the active site (Extended Data Figure 5). The diMerence in thermodynamic stability may therefore derive from additional folding garnered by glycine interactions with Apt-I, which are not required for Apt-II. Consistently, in some glycine riboswitch homologs, where Apt-I displays the higher aMinity interactions, the extra G-C base pair is found in Apt-I instead of Apt-II^21^. We speculate that an ensemble analysis of these homologs would yield an inverse finding of aptamer stability, where the population of Apt-I is higher than Apt-II in the absence of glycine and/or Mg^2+^. Interestingly, several studies have shown that RNA folding and compaction increases as the concentration of Mg^2+^ increases^25,33,34,81^, raising the intriguing possibility of enriching partially folded conformations by sampling at various Mg^2+^ concentrations.

Characterization of sparsely populated states, often the etiological agents of disease, has led to significant advancements in our understanding of misfolding-linked diseases^82,83^. Additionally, many disease-causing mutations operate at the ensemble level, where changes to the sequence result in abrogated populations of folds that can only be identified via an ensemble analysis^76^. Thus, it is important that work attempting to characterize RNA structure accounts for the highly heterogeneous nature of the biomolecule. Our work demonstrates that CryoEM can be used to generate sub-3Å RNA-only structures, provide information on RNA dynamics, and characterize low-population states in diMerent conditions, allowing one to visualize the remodeling of the configurational landscape under various conditions. With the continuing advances in data collection and processing rates, it may soon be possible to capture mid to high-resolution information on RNA folding pathways and misfolds. While huge advancements in protein fold prediction have occurred since the release of AlphaFold^84^, the RNA field has less high-resolution training data available, not to mention the significant impact buMer conditions have on RNA folding^2^. Collection of large cryoEM datasets may serve to address these challenges. The increasingly broad utilization of RNA within the fields of medicine and synthetic biology^85,86^ necessitates an improved understanding of RNA structure and dynamics.

## Extended Data

**Extended Data Table 1.** Cryo-EM data collection, refinement, and model statistics.

CryoEM data collection and processing	holo glyRS – glycine and Mg^2+^	glyRS – Mg^2+^ alone	glyRS - no ligand, no Mg^2+^
Voltage (kV)	300	300	300
Pixel size (Å)	0.823	0.823	0.823
Electron exposure (e-/Å^2^)	59	59	59
Defocus range	−0.6 to −2.4	−0.6 to −2.4	−0.6 to −2.4
Final micrographs used	5,338	5,819	4,312
Particle images (10 models)	384,275	725,637	430,437
Particle images (final)	215,269	207,411	
Resolution (FSC threshold 0.143)	2.86 Å	3.27 Å	
Map sharpening B-factor (Å^2^)	−51.8	−68.2	
			
**Refinement**			
Reference models	PDB: 6WLT, 3P49	Holo glyRS	
Model composition			
Atoms	7477	7443	
Protein residues	2	0	
RNA Residues	230	230	
Waters	11	7	
Mg^2+^	14	12	
CC_mask_	0.81	0.77	
r.m.s deviations			
Bond lengths (Å)	0.006	0.006	
Bond angles (°)	0.589	0.571	
Clash score	1.88	4.58	
MolProbity score	2.10	2.38	
Pucker outliers	0	0	

## Data Availability

The cryo-EM density maps and model coordinates have been deposited in the EM Data Bank (https://www.ebi.ac.uk/pdbe/emdb/) and the Protein Data Bank (https://www.rcsb.org) with accession codes EMD-48851, and 9N3I for the holo glycine riboswitch sample, and EMD-48852 and 9N3J for the Mg^2+^ alone sample. A previously published model Kappel *et al,* (2020) was used as a starting template (PDB code: 6WLT)^35^.

## References

[1] Simensen V. Experimental determination of Escherichia coli biomass composition for constraint-based metabolic modeling. PLoS One 17, (2022). 10.1371/journal.pone.0262450 PMC8794083 35085271

[2] Schneider B. When will RNA get its AlphaFold moment? Nucleic Acids Res 51, 9522–9532 (2023). 37702120 10.1093/nar/gkad726 PMC10570031

[3] Berman H. M. The Protein Data Bank. Nucleic Acids Res 28, 235 (2000). 10592235 10.1093/nar/28.1.235 PMC102472

[4] Hershkovitz E., Sapiro G., Tannenbaum A. & Williams L. D. Statistical Analysis of RNA Backbone. IEEE/ACM transactions on computational biology and bioinformatics / IEEE, ACM 3, 33 (2006). 10.1109/TCBB.2006.13 PMC2811324 17048391

[5] Woodson S. A. Compact intermediates in RNA folding: Annual Reviews in Biophysics. Annu Rev Biophys 39, 61 (2010). 20192764 10.1146/annurev.biophys.093008.131334 PMC6341483

[6] Prajapati J. D., Onuchic J. N. & Sanbonmatsu K. Y. Exploring the Energy Landscape of Riboswitches Using Collective Variables Based on Tertiary Contacts. J Mol Biol 434, 167788 (2022). 35963460 10.1016/j.jmb.2022.167788 PMC10042644

[7] Manz C. Exploring the energy landscape of a SAM-I riboswitch. J Biol Phys 47, 371–386 (2021). 34698957 10.1007/s10867-021-09584-7 PMC8603990

[8] Hennelly S. P., Novikova I. V. & Sanbonmatsu K. Y. The expression platform and the aptamer: cooperativity between Mg2+ and ligand in the SAM-I riboswitch. Nucleic Acids Res 41, 1922–1935 (2013). 23258703 10.1093/nar/gks978 PMC3562059

[9] Musier-Forsyth K., Rein A. & Hu W. S. Transcription start site choice regulates HIV-1 RNA conformation and function. Curr Opin Struct Biol 88, (2024). 10.1016/j.sbi.2024.102896 PMC11932513 39146887

[10] Fromm S. A. The translating bacterial ribosome at 1.55 Å resolution generated by cryo-EM imaging services. Nature Communications 2023 14:1 14, 1–9 (2023). 10.1038/s41467-023-36742-3 PMC9968351 36841832

[11] Li S., Palo M. Z., Zhang X., Pintilie G. & Zhang K. Snapshots of the second-step self-splicing of Tetrahymena ribozyme revealed by cryo-EM. Nature Communications 2023 14:1 14, 1–10 (2023). 10.1038/s41467-023-36724-5 PMC10020454 36928031

[12] Chen J. Ensemble cryo-EM reveals conformational states of the nsp13 helicase in the SARS-CoV-2 helicase replication-transcription complex. Nat Struct Mol Biol 29, 250–260 (2022). 35260847 10.1038/s41594-022-00734-6 PMC8935131

[13] Scheres S. H. W. Disentangling conformational states of macromolecules in 3D-EM through likelihood optimization. Nat Methods 4, 27–29 (2007). 17179934 10.1038/nmeth992

[14] Mandal M. A glycine-dependent riboswitch that uses cooperative binding to control gene expression. Science 306, 275–279 (2004). 15472076 10.1126/science.1100829

[15] Dhakal S. H., Panchapakesan S. S. S., Slattery P., Roth A. & Breaker R. R. Variants of the guanine riboswitch class exhibit altered ligand specificities for xanthine, guanine, or 20-deoxyguanosine. Proc Natl Acad Sci U S A 119, e2120246119 (2022). 35622895 10.1073/pnas.2120246119 PMC9295807

[16] Fuchs R. T., Grundy F. J. & Henkin T. M. The S(MK) box is a new SAM-binding RNA for translational regulation of SAM synthetase. Nat Struct Mol Biol 13, 226–233 (2006). 16491091 10.1038/nsmb1059

[17] Winkler W. C. & Breaker R. R. Genetic Control by Metabolite-Binding Riboswitches. ChemBioChem 4, 1024–1032 (2003). 14523920 10.1002/cbic.200300685

[18] Barrick J. E. & Breaker R. R. The distributions, mechanisms, and structures of metabolite-binding riboswitches. Genome Biol 8, 1–19 (2007). 10.1186/gb-2007-8-11-r239 PMC2258182 17997835

[19] Crum M., Ram-Mohan N. & Meyer M. M. Regulatory context drives conservation of glycine riboswitch aptamers. PLoS Comput Biol 15, (2019). 10.1371/journal.pcbi.1007564 PMC6944388 31860665

[20] Sherlock M. E. Architectures and complex functions of tandem riboswitches. RNA Biol 19, 1059–1076 (2022). 36093908 10.1080/15476286.2022.2119017 PMC9481103

[21] Torgerson C. D., Hiller D. A. & Strobel S. A. The asymmetry and cooperativity of tandem glycine riboswitch aptamers. RNA 26, 564–580 (2020). 31992591 10.1261/rna.073577.119 PMC7161355

[22] Kwon M. & Strobel S. A. Chemical basis of glycine riboswitch cooperativity. RNA 14, 25 (2008). 18042658 10.1261/rna.771608 PMC2151043

[23] Butler E. B., Xiong Y., Wang J. & Strobel S. A. Structural Basis of Cooperative Ligand Binding by the Glycine Riboswitch. Chem Biol 18, 293–298 (2011). 21439473 10.1016/j.chembiol.2011.01.013 PMC3076126

[24] Huang L., Serganov A. & Patel D. J. Structural Insights into Ligand Recognition by a Sensing Domain of the Cooperative Glycine Riboswitch. Mol Cell 40, 774–786 (2010). 21145485 10.1016/j.molcel.2010.11.026 PMC3726718

[25] Lipfert J. Structural transitions and thermodynamics of a glycine-dependent riboswitch from Vibrio cholerae. J Mol Biol 365, 1393–1406 (2007). 17118400 10.1016/j.jmb.2006.10.022 PMC1941672

[26] Babina A. M., Lea N. E. & Meyer M. M. In vivo behavior of the tandem glycine riboswitch in Bacillus subtilis. mBio 8, (2017). 10.1128/mBio.01602-17 PMC5666159 29089431

[27] RuM K. M. & Strobel S. A. Ligand binding by the tandem glycine riboswitch depends on aptamer dimerization but not double ligand occupancy. RNA 20, 1775 (2014). 25246650 10.1261/rna.047266.114 PMC4201829

[28] Sherman E. M., Esquiaqui J., Elsayed G. & Ye J. D. An energetically beneficial leader-linker interaction abolishes ligand-binding cooperativity in glycine riboswitches. RNA 18, 496–507 (2012). 22279151 10.1261/rna.031286.111 PMC3285937

[29] Kladwang W., Chou F. C. & Das R. Automated RNA structure prediction uncovers a kink-turn linker in double glycine riboswitches. J Am Chem Soc 134, 1404–1407 (2012). 22192063 10.1021/ja2093508

[30] Crum M., Ram-Mohan N. & Meyer M. M. Regulatory context drives conservation of glycine riboswitch aptamers. PLoS Comput Biol 15, e1007564 (2019). 31860665 10.1371/journal.pcbi.1007564 PMC6944388

[31] Chu V. B., Bai Y., Lipfert J., Herschlag D. & Doniach S. A repulsive field: advances in the electrostatics of the ion atmosphere. Curr Opin Chem Biol 12, 619–625 (2008). 19081286 10.1016/j.cbpa.2008.10.010 PMC2976615

[32] Draper D. E., Grilley D. & Soto A. M. Ions and RNA folding. Annu Rev Biophys Biomol Struct 34, 221–243 (2005). 15869389 10.1146/annurev.biophys.34.040204.144511

[33] Hennelly S. P., Novikova I. V. & Sanbonmatsu K. Y. The expression platform and the aptamer: cooperativity between Mg2+ and ligand in the SAM-I riboswitch. Nucleic Acids Res 41, 1922 (2013). 23258703 10.1093/nar/gks978 PMC3562059

[34] Lipfert J., Sim A. Y. L., Herschlag D. & Doniach S. Dissecting electrostatic screening, specific ion binding, and ligand binding in an energetic model for glycine riboswitch folding. RNA 16, 708–719 (2010). 20194520 10.1261/rna.1985110 PMC2844619

[35] Kappel K. Accelerated cryo-EM-guided determination of three-dimensional RNA-only structures. Nature Methods 2020 17:7 17, 699–707 (2020). 32616928 10.1038/s41592-020-0878-9 PMC7386730

[36] Roy S., Hennelly S. P., Lammert H., Onuchic J. N. & Sanbonmatsu K. Y. Magnesium controls aptamer-expression platform switching in the SAM-I riboswitch. Nucleic Acids Res 47, 3158–3170 (2019). 30605518 10.1093/nar/gky1311 PMC6451092

[37] Suloway C. Automated molecular microscopy: the new Leginon system. J Struct Biol 151, 41–60 (2005). 15890530 10.1016/j.jsb.2005.03.010

[38] Punjani A., Rubinstein J. L., Fleet D. J. & Brubaker M. A. cryoSPARC: algorithms for rapid unsupervised cryo-EM structure determination. Nature Methods 2017 14:3 14, 290–296 (2017). 10.1038/nmeth.4169 28165473

[39] Punjani A., Zhang H. & Fleet D. J. Non-uniform refinement: adaptive regularization improves single-particle cryo-EM reconstruction. Nature Methods 2020 17:12 17, 1214–1221 (2020). 10.1038/s41592-020-00990-8 33257830

[40] Punjani A. & Fleet D. J. 3D variability analysis: Resolving continuous flexibility and discrete heterogeneity from single particle cryo-EM. J Struct Biol 213, 107702 (2021). 33582281 10.1016/j.jsb.2021.107702

[41] Emsley P., Lohkamp B., Scott W. G. & Cowtan K. Features and development of Coot. Acta Crystallogr D Biol Crystallogr 66, 486–501 (2010). 20383002 10.1107/S0907444910007493 PMC2852313

[42] Croll T. I. ISOLDE: A physically realistic environment for model building into low-resolution electron-density maps. Acta Crystallogr D Struct Biol 74, 519–530 (2018). 29872003 10.1107/S2059798318002425 PMC6096486

[43] Adams P. D. The Phenix software for automated determination of macromolecular structures. Methods 55, 94–106 (2011). 21821126 10.1016/j.ymeth.2011.07.005 PMC3193589

[44] Goddard T. D. UCSF ChimeraX: Meeting modern challenges in visualization and analysis. Protein Science 27, 14–25 (2018). 28710774 10.1002/pro.3235 PMC5734306

[45] Prajapati J. D., Onuchic J. N. & Sanbonmatsu K. Y. Exploring the Energy Landscape of Riboswitches Using Collective Variables Based on Tertiary Contacts. J Mol Biol 434, 167788 (2022). 35963460 10.1016/j.jmb.2022.167788 PMC10042644

[46] Zgarbová M. Refinement of the Cornell et al. Nucleic Acids Force Field Based on Reference Quantum Chemical Calculations of Glycosidic Torsion Profiles. J Chem Theory Comput 7, 2886–2902 (2011). 21921995 10.1021/ct200162x PMC3171997

[47] Tian C. Ff19SB: Amino-Acid-Specific Protein Backbone Parameters Trained against Quantum Mechanics Energy Surfaces in Solution. J Chem Theory Comput 16, 528–552 (2020). 31714766 10.1021/acs.jctc.9b00591 PMC13071887

[48] Mamatkulov S. & Schwierz N. Force fields for monovalent and divalent metal cations in TIP3P water based on thermodynamic and kinetic properties. J Chem Phys 148, (2018). 10.1063/1.5017694 29471634

[49] Grotz K. K. & Schwierz N. Optimized Magnesium Force Field Parameters for Biomolecular Simulations with Accurate Solvation, Ion-Binding, and Water-Exchange Properties in SPC/E, TIP3P-fb, TIP4P/2005, TIP4P-Ew, and TIP4P-D. J Chem Theory Comput 18, 526–537 (2022). 34881568 10.1021/acs.jctc.1c00791 PMC8757469

[50] Essmann U. A smooth particle mesh Ewald method. J Chem Phys 103, 8577–8593 (1995).

[51] Hess B., Bekker H., Berendsen H. J. C. & Fraaije J. G. E. M. LINCS: A Linear Constraint Solver for Molecular Simulations. J Comput Chem 18, 14631472 (1997).

[52] Hess B., Kutzner C., van der Spoel D. & Lindahl E. GROMACS 4: Algorithms for Highly EMicient, Load-Balanced, and Scalable Molecular Simulation. J Chem Theory Comput 4, 435–447 (2008). 26620784 10.1021/ct700301q

[53] Tribello G. A., Bonomi M., Branduardi D., Camilloni C. & Bussi G. PLUMED 2: New feathers for an old bird. Comput Phys Commun 185, 604–613 (2014).

[54] Barducci A., Bussi G. & Parrinello M. Well-tempered metadynamics: A smoothly converging and tunable free-energy method. Phys Rev Lett 100, 020603 (2008). 18232845 10.1103/PhysRevLett.100.020603

[55] Raiteri P., Laio A., Gervasio F. L., Micheletti C. & Parrinello M. EMicient reconstruction of complex free energy landscapes by multiple walkers metadynamics. J Phys Chem B 110, 3533–3539 (2006). 16494409 10.1021/jp054359r

[56] Prajapati J. D., Fernández Solano C. J., Winterhalter M. & Kleinekathöfer U. Characterization of Ciprofloxacin Permeation Pathways across the Porin OmpC Using Metadynamics and a String Method. J Chem Theory Comput 13, 4553–4566 (2017). 28816443 10.1021/acs.jctc.7b00467

[57] Golla V. K., Prajapati J. D., Joshi M. & Kleinekathöfer U. Exploration of Free Energy Surfaces Across a Membrane Channel Using Metadynamics and Umbrella Sampling. J Chem Theory Comput 16, 2751–2765 (2020). 32167296 10.1021/acs.jctc.9b00992

[58] Tiwary P. & Parrinello M. A time-independent free energy estimator for metadynamics. J Phys Chem B 119, 736–742 (2015). 25046020 10.1021/jp504920s

[59] Whitford P. C. An all-atom structure-based potential for proteins: Bridging minimal models with all-atom empirical forcefields. Proteins: Structure, Function, and Bioinformatics 75, 430–441 (2009). 10.1002/prot.22253 PMC3439813 18837035

[60] Van Der Spoel D. GROMACS: Fast, flexible, and free. J Comput Chem 26, 1701–1718 (2005). 16211538 10.1002/jcc.20291

[61] Noel J. K. SMOG 2: A Versatile Software Package for Generating Structure-Based Models. PLoS Comput Biol 12, e1004794 (2016). 26963394 10.1371/journal.pcbi.1004794 PMC4786265

[62] Ratje A. H. Head swivel on the ribosome facilitates translocation by means of intra-subunit tRNA hybrid sites. Nature 468, 713–716 (2010). 21124459 10.1038/nature09547 PMC3272701

[63] Whitford P. C. Excited states of ribosome translocation revealed through integrative molecular modeling. Proceedings of the National Academy of Sciences 108, 18943–18948 (2011). 10.1073/pnas.1108363108 PMC3223460 22080606

[64] Kim D. N. Cryo_fit: Democratization of flexible fitting for cryo-EM. J Struct Biol 208, 1–6 (2019). 31279069 10.1016/j.jsb.2019.05.012 PMC7112765

[65] Humphrey W., Dalke A. & Schulten K. VMD: Visual molecular dynamics. J Mol Graph 14, 33–38 (1996). 8744570 10.1016/0263-7855(96)00018-5

[66] Pettersen E. F. UCSF Chimera—A visualization system for exploratory research and analysis. J Comput Chem 25, 1605–1612 (2004). 15264254 10.1002/jcc.20084

[67] Deng J. RNA structure determination: From 2D to 3D. Fundamental Research 3, 727–737 (2023). 38933295 10.1016/j.fmre.2023.06.001 PMC11197651

[68] Wang H. W. & Wang J. W. How cryo-electron microscopy and X-ray crystallography complement each other. Protein Sci 26, 32 (2017). 27543495 10.1002/pro.3022 PMC5192981

[69] Zheng H. CheckMyMetal: a macromolecular metal-binding validation tool. Acta Crystallogr D Struct Biol 73, 223 (2017). 28291757 10.1107/S2059798317001061 PMC5349434

[70] Jain S., Richardson D. C. & Richardson J. S. Computational Methods for RNA Structure Validation and Improvement. Methods Enzymol 558, 181–212 (2015). 26068742 10.1016/bs.mie.2015.01.007

[71] Winkler W. C. Metabolic monitoring by bacterial mRNAs. Arch Microbiol 183, 151–159 (2005). 15750802 10.1007/s00203-005-0758-9

[72] Winkler W. C. & Breaker R. R. Regulation of bacterial gene expression by riboswitches. Annu Rev Microbiol 59, 487–517 (2005). 16153177 10.1146/annurev.micro.59.030804.121336

[73] Yabukarski F. Ensemble-function relationships to dissect mechanisms of enzyme catalysis. Sci Adv 8, (2022). 10.1126/sciadv.abn7738 PMC9565801 36240280

[74] Tesei G. Conformational ensembles of the human intrinsically disordered proteome. Nature 626, 897–904 (2024). 38297118 10.1038/s41586-023-07004-5

[75] Bonilla S. L., Jones A. N. & Incarnato D. Structural and biophysical dissection of RNA conformational ensembles. Curr Opin Struct Biol 88, 102908 (2024). 39146886 10.1016/j.sbi.2024.102908

[76] Ganser L. R., Kelly M. L., Herschlag D. & Al-Hashimi H. M. The roles of structural dynamics in the cellular functions of RNAs. Nature Reviews Molecular Cell Biology 2019 20:8 20, 474–489 (2019). 31182864 10.1038/s41580-019-0136-0 PMC7656661

[77] Helmling C. Life times of metastable states guide regulatory signaling in transcriptional riboswitches. Nature Communications 2018 9:1 9, 1–9 (2018). 10.1038/s41467-018-03375-w PMC5838219 29507289

[78] Helmling C. NMR Structural Profiling of Transcriptional Intermediates Reveals Riboswitch Regulation by Metastable RNA Conformations. J Am Chem Soc 139, 2647–2656 (2017). 28134517 10.1021/jacs.6b10429

[79] Spitale R. C. & Incarnato D. Probing the dynamic RNA structurome and its functions. Nat Rev Genet 24, 178 (2022). 36348050 10.1038/s41576-022-00546-w PMC9644009

[80] Chauvier A. Transcriptional pausing at the translation start site operates as a critical checkpoint for riboswitch regulation. Nature Communications 2016 8:1 8, 1–12 (2017). 10.1038/ncomms13892 PMC5234074 28071751

[81] Tan Z. J. & Chen S. J. Ion-Mediated RNA Structural Collapse: EMect of Spatial Confinement. Biophys J 103, 827 (2012). 22947944 10.1016/j.bpj.2012.06.048 PMC3443784

[82] Karamanos T. K., Kalverda A. P., Thompson G. S. & Radford S. E. Mechanisms of amyloid formation revealed by solution NMR. Prog Nucl Magn Reson Spectrosc 88–89, 86–104 (2015). 10.1016/j.pnmrs.2015.05.002 PMC4568309 26282197

[83] Alderson T. R. & Kay L. E. Unveiling invisible protein states with NMR spectroscopy. Curr Opin Struct Biol 60, 39–49 (2020). 31835059 10.1016/j.sbi.2019.10.008

[84] Jumper J. Highly accurate protein structure prediction with AlphaFold. Nature 596, 583–589 (2021). 34265844 10.1038/s41586-021-03819-2 PMC8371605

[85] McKeague M., Wong R. S. & Smolke C. D. Opportunities in the design and application of RNA for gene expression control. Nucleic Acids Res 44, 2987–2999 (2016). 26969733 10.1093/nar/gkw151 PMC4838379

[86] Childs-Disney J. L. & Disney M. D. Approaches to Validate and Manipulate RNA Targets with Small Molecules in Cells. Annu Rev Pharmacol Toxicol 56, 123 (2016). 26514201 10.1146/annurev-pharmtox-010715-103910 PMC4876813

